# Reasons for Neonatal Presentations to Pediatric Emergency Departments in Catania: Multicentric Cross‐Sectional Analysis and Exhaustive Review of the Literature

**DOI:** 10.1111/birt.12877

**Published:** 2024-09-24

**Authors:** Raffaele Falsaperla, Mariaclaudia Meli, Vincenzo Sortino, Silvia Marino, Lucia Tardino, Gian Luca Trobia, Massimo Barbagallo, Bruna Scalia, the Sicilian Group of the Italian Society of Neonatology (SIN)

**Affiliations:** ^1^ Neonatal Intensive Care Unit, Azienda Ospedaliero Universitaria Policlinico “G. Rodolico—San Marco” University of Catania Catania Italy; ^2^ Pediatric and Pediatric Emergency Department Azienda Ospedaliero Universitaria Policlinico “G. Rodolico—San Marco”, University of Catania Catania Italy; ^3^ Pediatric and Pediatric Emergency Room Unit "Cannizzaro" Emergency Hospital Catania Catania Italy; ^4^ Department of Paediatrics Azienda Ospedaliera di Rilievo Nazionale e di Alta Specializzazione "Garibaldi" Catania Italy

**Keywords:** jaundice, newborns, pediatric emergency department, pediatric primary care

## Abstract

**Introduction:**

This study aimed to characterize neonatal admissions to pediatric emergency departments (PEDs) in Catania, to analyze the primary pediatric conditions leading to these admissions, and to explore the association between the demographic characteristics of the population and the severity of their presentations.

**Materials and Methods:**

A retrospective analysis was conducted on neonates (aged <28 days) admitted to three PEDs in Catania between January 2015 and December 2019. Additionally, a comprehensive review of the literature on this topic was performed.

**Results:**

A total of 5183 neonates presented during the study period, with a median age of 14 days at admission. The top three diagnoses were neonatal jaundice (15%), abdominal discomfort (12%), and upper airway inflammation (11%). The majority of cases were classified as non‐urgent (green) at triage (59%). Overall, 1296 patients (25%) required hospitalization; 95% of those assigned a yellow triage color at admission required hospitalization. Only 33% of hospitalized patients were referred by parents, while the majority were referred by primary care pediatricians. The highest number of admissions occurred in August, while the peak in hospitalizations was in February.

**Conclusions:**

The majority of neonatal PED admissions are for non‐acute conditions that do not require immediate medical attention. This concerning trend leads to increased workloads for PED staff, higher healthcare costs, and potential risks to neonates. Possible causes include insufficient caregiver knowledge, inadequate parental education, and suboptimal transition from hospital to primary care pediatric services.

## Graphical abstract

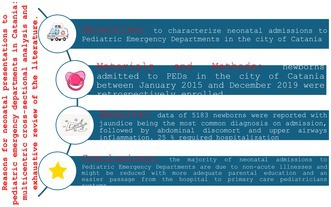

## 1 Introduction

In recent decades, both the literature and clinical practice have raised concerns about the increasing number of neonatal admissions to pediatric emergency departments (PEDs) [1, 2]. This trend likely reflects a shift towards earlier discharge of neonates in the postpartum period. While shorter hospital stays can enhance maternal–infant bonding, promote parental involvement, and optimize healthcare resource allocation, they may also contribute to the rising number of PED visits [3].

The neonatal period is a particularly vulnerable time, marked by signs and symptoms that are often vague and non‐specific, which can be indicative of either benign conditions or serious diseases. This uncertainty, coupled with the anxieties of inexperienced parents who have been discharged early, likely explains the increased attendance of neonates at PEDs [4, 5, 6, 7].

In Italy, the healthcare system provides free public care to all patients, including healthy newborns who stay with their mothers in Obstetrics and Gynecology (Ob/Gyn) departments. These newborns are discharged by a neonatologist after 48 h and referred directly to primary care pediatricians. However, this transition is often fraught with delays, as the offices responsible for processing these referrals are frequently closed or overcrowded, and primary care pediatricians are often at full capacity, unable to accept additional patients. This gap in care leads many parents to bring their newborns to PEDs for issues that could be managed elsewhere, thereby increasing healthcare costs and exposing neonates to additional risks, such as nosocomial infections [8, 9].

Factors such as maternal age, parity, lack of family support, race, education level, and absence of prenatal care are also associated with more frequent PED admissions for non‐urgent reasons [8, 9]. Despite these concerns, few studies have thoroughly evaluated the incidence and reasons for neonatal admissions to PEDs [8, 9, 10, 11]. Our comprehensive review of the literature across several medical databases revealed that most PED visits are attributable to non‐serious conditions and insufficient caregiver knowledge about the neonatal period. Many of these visits could have been avoided with better pre‐ and post‐natal education and more robust primary care support [10, 11].

Interestingly, none of the available data specifically address the situation in Italy. To fill this knowledge gap, we collected demographic and clinical data of newborns admitted to three different PEDs in Catania from January 2015 to December 2019. Conducted on behalf of the Sicilian Group of the Italian Society of Neonatology (SIN), this study aims to identify the reasons for neonatal presentations to PEDs in our city, assess the severity of their conditions, analyze hospitalization rates, and explore the factors associated with these outcomes. Our secondary goal is to understand the determinants of neonatal PED presentations in our region, with the aim of identifying opportunities to reduce unnecessary visits and their associated drawbacks [12].

## 2 Materials and Methods

### 2.1 Study Design and Period

This study is a retrospective, multicenter cohort analysis conducted over a 5‐year period, from January 2015 to December 2019.

### 2.2 Study Sites

Data were collected from the following three major hospitals in Catania, Italy:

1. San Marco University Hospital
2. Garibaldi First Aid Hospital
3. Cannizzaro First Aid Hospital

These PEDs are part of tertiary care hospitals in Catania and collectively serve a pediatric population of approximately 164,000 children aged 0–14 years. The PEDs provide emergency care 24 h a day, 7 days a week.

### 2.3 Inclusion Criteria

The study included neonates who:

1. were between 0 and 28 days old
2. were admitted to any of the aforementioned PEDs
3. presented for any reason

### 2.4 Exclusion Criteria

Exclusion criteria were:

1. patients older than 28 days
2. Cases with insufficient medical records

### 2.5 Data Source and Collection

For each patient, all electronic emergency room reports and any relevant paper medical records were manually reviewed. The severity of each case was assessed based on the triage color code assigned at admission, clinical data recorded upon arrival, and the discharge diagnosis. Clinical and demographic data were collected for each patient, including gender, age, weight, height, vital signs, month of admission, reason for admission, discharge status or hospitalization, duration of hospital stay if admitted, and the triage color code assigned according to the Italian triage system (Tables 1, 2, 3).

**TABLE 1:** Demographic features and reasons for neonatal admissions to Catania's PEDs between January 2015 and December 2019.

	San Marco Hospital (*n*, %)	Cannizzaro Hospital (*n*, %)	Garibaldi Hospital (*n*, %)	Total neonatal patients (*n*, %)
Males	414 (54)	411 (55)	2054 (56)	2879
Females	353 (46)	337 (45)	1614 (44)	2304
Green color triage	437 (57)	479 (64)	2127 (58)	3043
Yellow color triage	330 (43)	269 (46)	1541 (42)	2140
Self‐referral	595 (18)	501 (16)	2169 (66)	3265 (63%)
Pediatrician‐referral	429 (22)	516 (27)	973 (51)	1918 (37%)
Hospitalization	192 (25)	187 (25)	917 (25)	1296 (25%)
Self‐referred hospitalized patients	79	80	264	423 (8%)
Pediatrician‐referred hospitalized patients	113	106	654	873 (17%)
Discharge	575 (75)	561 (75)	2751 (75)	3887
Causes for ED visits				
Abdominal colic	67 (8.7)	87 (11.5)	477 (13)	631 (12)
Regurgitation	74 (9.6)	89 (11.9)	202 (5.5)	365 (7)
Ear infection	90 (11.6)	23 (3)	55 (1.5)	168 (3)
Cyanosis	73 (9.6)	53 (7)	110 (3)	236 (5)
Upper airways inflammation	110 (14.4)	110 (14.4)	330 (9)	550 (11)
Jaundice	28 (3.7)	73 (9.7)	661 (18)	762 (15)
Bronchiolitis	38 (5)	64 (8.4)	183 (5)	285 (6)
Rash	30 (4)	32 (4.2)	109 (3)	171 (4)
Conjunctivitis	15 (2)	8 (1)	36 (1)	59 (2)
Temperature	37 (4.9)	36 (4.7)	147 (4)	220 (5)
Enteritis	35 (4.6)	53 (7)	220 (6)	308 (6)
Others	170 (22)	120 (16)	1138 (31)	1428 (28)

The Italian triage system is a professional emergency assessment tool used to prioritize patients based on the severity of their condition at the time of admission to a PED. Patients are categorized into four color codes: white, green, yellow, and red, with severity increasing from white to red. Yellow and red codes indicate urgent cases, while white and green codes are non‐urgent [13].

**FIGURE 1:**
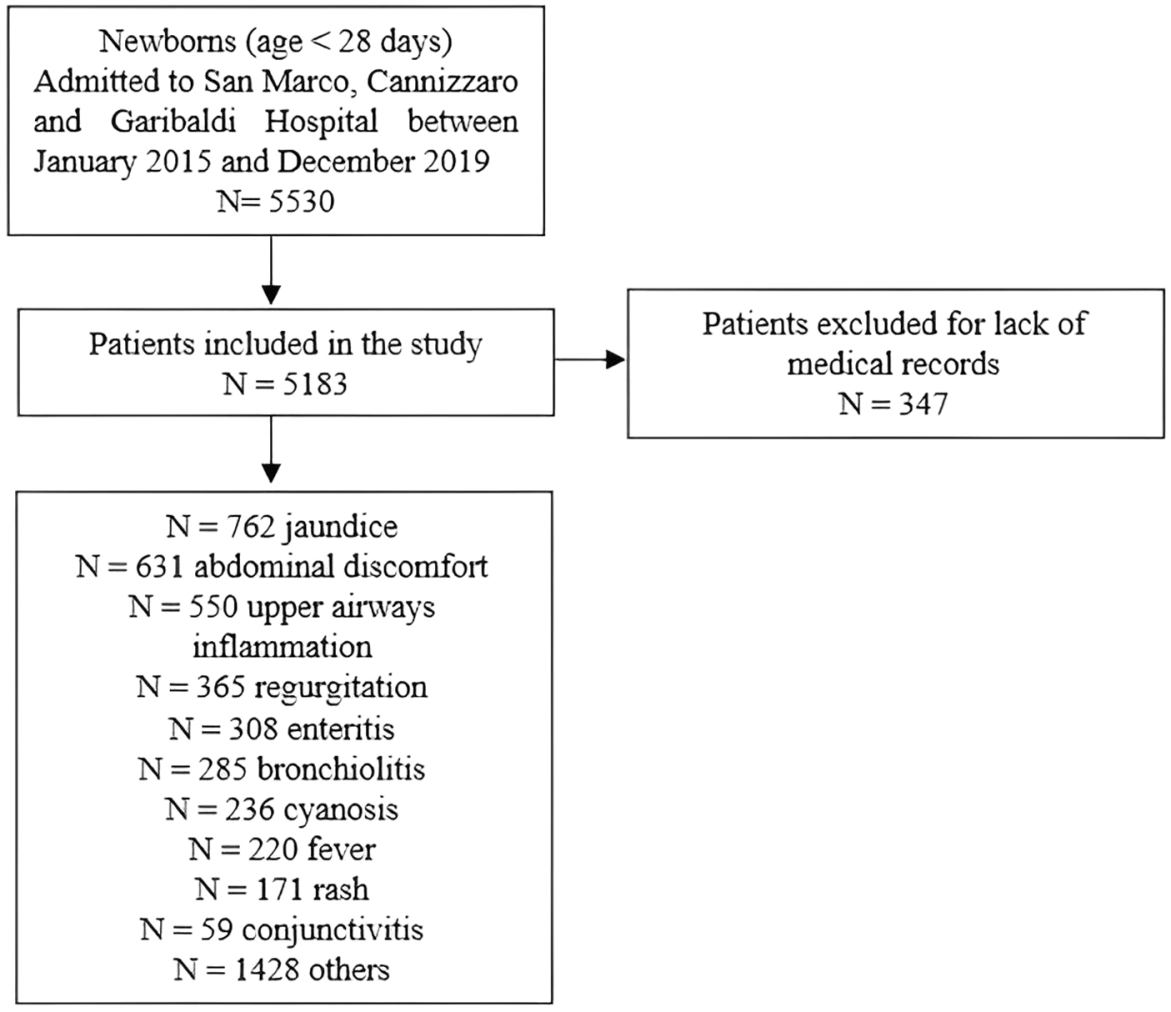
Selection process of the patients included.

Additionally, the number of admissions and the corresponding triage color codes were recorded for each month of the study period to determine any correlation between the severity of cases and the triage color assigned, as well as to identify any seasonal trends in admission rates. The selection process is illustrated in Figure 1.

### 2.6 Ethics Approval

The study received approval from the ethics committee of our institution. All research procedures were conducted in accordance with relevant guidelines and regulations. Informed consent was obtained from the parents of all participating patients. The study adhered to the ethical principles outlined in the Declaration of Helsinki [14].

### 2.7 Exhaustive Review of the Literature

**FIGURE 2:**
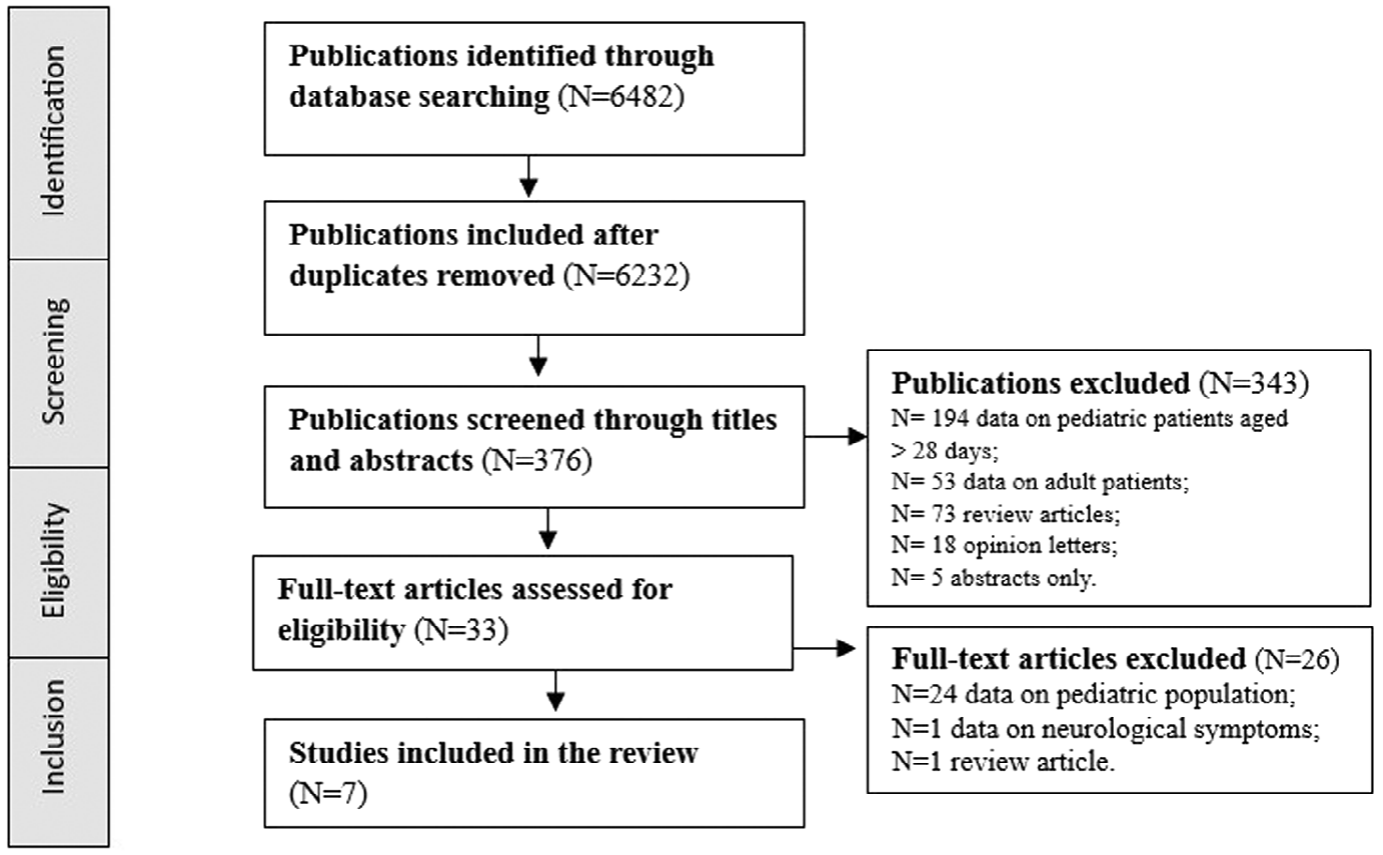
The PRISMA diagram selection process of the studies included. PRISMA preferred reporting items for systematic review and meta‐analysis.

In addition, we conducted an exhaustive review of the literature on neonatal admissions to PEDs using various medical electronic databases, including the Cochrane Library, Medline, PubMed Central, Scopus, and Web of Science. This review adhered to the Preferred Reporting Items for Systematic Reviews and Meta‐Analyses (PRISMA) guidelines (Figure 2).

The search terms employed were as follows: “newborns” AND “pediatric emergency department,” “neonates” AND “pediatric emergency departments,” and “neonates” AND “reasons admission pediatric emergency department.” The search yielded 22 articles published from 2012 to the present. Of these, 15 articles were deemed irrelevant and subsequently excluded, resulting in the inclusion of 8 articles (Table 4). All selected studies focused on neonatal populations. The only filters applied were for English‐language publications and studies involving human subjects. Notably, none of the previously published studies reported on characteristics of the Italian neonatal population.

### 2.8 Statistical Analysis

Quantitative data (e.g., newborns' age) were expressed as means, while qualitative data (e.g., gender, emergency triage color, reason for the visit, discharge or hospitalization status, and duration of hospitalization) were presented as percentages. The Kolmogorov–Smirnov one‐sample test was used to assess the normal distribution of quantitative variables.

## 3 Results

### 3.1 PED Presentations

Analysis of the data revealed that between 2015 and 2020, there were 62,886 admissions to the PED at San Marco Hospital (Observational Center [OC] 1), 184,224 admissions at Garibaldi First Aid Hospital (OC 2), and 53,692 admissions at Cannizzaro First Aid Hospital (OC 3), resulting in a total of 300,802 PED visits across the three hospitals. Of these, 32,519 (11%) resulted in hospitalization.

During the study period, 767 neonates were admitted to the PED in OC 1, 748 in OC 2, and 3668 in OC 3, totaling 5183 neonatal admissions across the PEDs in Catania. Among these, 3472 neonates (66.9%) were born at one of the three hospitals included in the study. Remarkably, with a total of 14,153 births in the Catania area during the study period, more than one‐third (36.6%) of newborns were referred to a PED after discharge.

### 3.2 Reasons for Neonatal Presentations

The most common reasons for neonatal admission are detailed in Table 1. The leading cause was jaundice (762 newborns, 15%), followed by abdominal discomfort (631 newborns, 12%), inflammation of the upper airways (550 newborns, 11%), oral regurgitation (365 newborns, 7%), enteritis (308 newborns, 6%), bronchiolitis (285 newborns, 6%), cyanosis (236 newborns, 5%), fever (220 newborns, 5%), rash (171 newborns, 4%), ear infection (168 newborns, 3%), and conjunctivitis (59 newborns, 2%). Additionally, 1428 newborns (28%) were admitted for various other, less common reasons, including trauma, post‐discharge general visits, constipation, insect stings, dehydration or loss of appetite, and brief resolved unexplained events (BRUE) (Table 1).

### 3.3 Factors Associated With Neonatal Presentations and Admissions

The median age at admission was 14 days, with a slight male predominance (55% male vs. 45% female), resulting in a male‐to‐female ratio of 1.2:1 (Table 1). Notably, the highest number of admissions occurred during the second week of life, while the peak hospitalization rate was observed during the fourth week (Table 2).

**TABLE 2:** Number of neonatal admissions to PEDs per week of life.

Week of life	OC1 (*N* %)	OC2 (*N* %)	OC3 (*N* %)	*N* of patients	Hospitalization (%)	Method of referral (*N* of patients and %)
1st	77 (10)	955 (27)	94 (14)	1126	195 (17)	Self, *N* = 900 (80%) Pediatrician, *N* = 226 (20%)
2nd	228 (30)	1059 (29)	178 (26)	1465	370 (25)	Self, *N* = 1042 (71, 2%) Pediatrician, *N* = 423 (28, 8%)
3rd	244 (32)	827 (22)	182 (27)	1253	336 (27)	Self, *N* = 664 (53%) Pediatrician, *N* = 589 (47%)
4th	218 (28)	827 (22)	298 (31)	1253	398 (32)	Self, *N* = 476 (38%) Pediatrician, *N* = 777 (62%)

**TABLE 3:** Number of neonatal PEDs admissions per month.

	San Marco Hospital *N* (%)	Cannizzaro Hospital *N* (%)	Garibaldi Hospital *N* %
January	84 (11)	71 (9.5)	367 (10)
February	69 (9)	67 (9)	311 (8.5)
March	69 (9)	69 (9.2)	330 (9)
April	38 (5)	43 (5.8)	257 (7)
May	38 (5)	52 (7)	238 (6.5)
June	69 (9)	63 (8.5)	348 (9,5)
July	77 (10)	52 (7)	367 (10)
August	84 (11)	88 (11.7)	403 (11)
September	69 (9)	71 (9.5)	311 (8.5)
October	46 (6)	66 (8.8)	257 (7)
November	53 (7)	69 (9.2)	220 (6)
December	69 (9)	45 (6)	257 (7)

**TABLE 4:** Exhaustive summary of the demographics data and reasons for visit among other studies.

Authors	Country	Purpose	Type of study	*N*. of patients	% of neonates admitted	Mean age or week of life	M/F ratio	Reasons for admission	Hospitalization
Calado et al. (2009)	Portugal	Characterization of newborn visits to PEDs	Retrospective	540	1.5%	14 days	53.1%/46.9%	‐Jaundice (14.5%) ‐Excessive crying (13.6%) ‐Rash (7.3%) ‐Nasal congestion (6.7%) ‐Cough (5.9%)	13%
Claudet et al. (2012)	France	Characterization of newborn visits to PEDs	Prospective	578	N/A	17 days	53%/47%	‐Gastrointestinal problems (25%) ‐Respiratory issues (21%) ‐Crying (12%) ‐Fever (11%) ‐Trauma (8%)	47%
Lee et al. (2014)	USA	Characterization of newborn visits to PEDs and examine variations by race	Retrospective	1.923.245	N/A	34% first week; 24% second week; 23% third week; 18% fourth week	N/A	‐Moderate infection (18.6%) ‐Benign gastrointestinal problems (14%) ‐Potential serious newborn problems (12.9%) ‐Jaundice (12%) ‐Routine infant care (11.6%)	N/A
Flanagan et al. (2014)	UK	Characterization of newborn visits to PEDs	Retrospective	208	N/A	N/A	N/A	‐Feeding difficulty (36%) ‐Respiratory infections (14%) ‐Jaundice (10%)	24%
Batu et al. (2015)	Turkey	Characterization of newborn visits to PEDs	Prospective	531	1.9%	14.1 ± 8.3	57.3%/42.7%	‐Jaundice (23.4%) ‐Irritability (9.5%) ‐Vomiting (7.1%)	23.2%
Yang et al. (2017)	Korea	Characterization of urgent versus non—urgent newborn visits to PEDs	Retrospective	1008	1.29%	9.8 days ±6.6	53%/46%	‐Jaundice (25.7%) ‐Fever (24.5%)	67%
Ferreira et al. (2018)	Portugal	Characterization of newborn visits to PEDs	Retrospective	378	1%	32% first week;27% second week; 23% third week; 18% fourth week	53.4%/46.6%	‐Gastrointestinal symptoms (33.8%) ‐Rash (21.4%) ‐Jaundice (16.2%)	16.9%
Lim et al. (2022)	Singapore		Retrospective	1200	N/A	79.4% in first and second week; 20.6% in the second and third week	51.7%/48.3%	‐Jaundice (66.8%) ‐Fever (14.6%) ‐Others (10.6%) ‐Well baby (5.8%) ‐Respiratory infections (5.3%)	57.7%
Falsaperla et al. (2023) *Present study*	Italy		Retrospective	5183	3%	22% in the first week; 29% in the second week; 24% in the third and fourth week	55%/45%	‐Jaundice (15%), ‐Abdominal discomfort (12%) ‐Upper airways inflammation (11%) ‐Regurgitation (7%) ‐Enteritis (6%)	25%

Abbreviations: D, days; F, female; M, male; *N*, number; N/A, not available; USA, United States of America; UK, United Kingdom.

**FIGURE 3:**
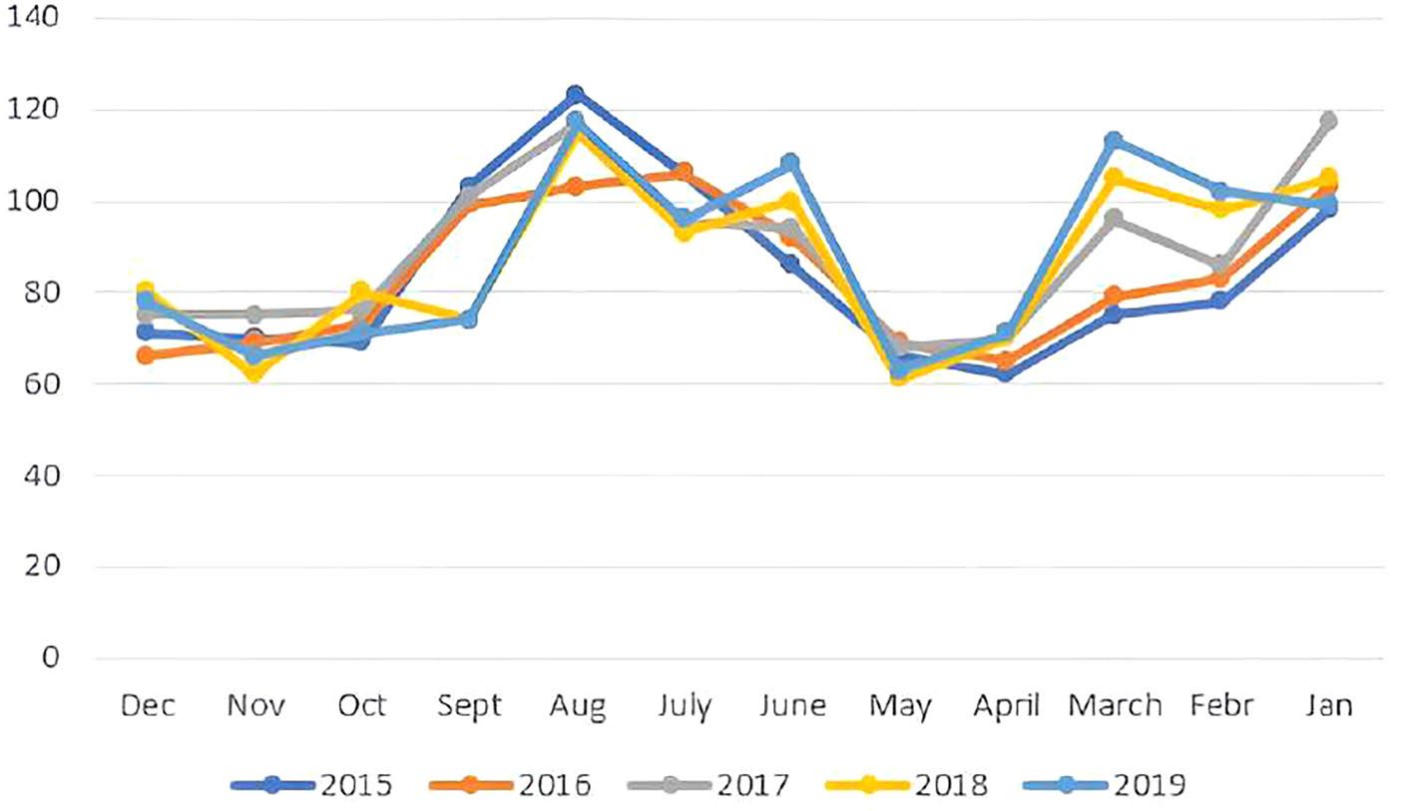
Trends of neonatal PEDs admission per month. [Colour figure can be viewed at wileyonlinelibrary.com]

As shown in Table 1, no neonate was assigned a “white” or “red” triage code. The majority, 3043 (59%), were assigned a “green” code, and 2140 (41%) received a “yellow” code. Overall, 1296 neonates (25%) required hospitalization, while 3888 (75%) were discharged home. Interestingly, 873 (67%) of the hospitalized neonates were referred by a pediatrician, while 423 (33%) were brought in by their parents. All neonates assigned a “yellow” triage code required hospitalization, with 95% of hospitalized newborns receiving this triage color at admission. The highest number of emergency department (ED) admissions occurred in August, whereas the peak in hospitalizations was recorded in February (Figure 3).

In order to contribute to the existing literature, we collected data on the individuals who brought the patients to the PEDs. The majority of neonates (63%) were brought in by their parents, while 37% were referred by primary care pediatricians. Notably, 75% of neonates admitted to PEDs during the first 2 weeks of life had not been under the care of a primary care pediatrician. This percentage dropped to 53% in the third week of life and to 38% in the fourth week.

## 4 Discussion

Neonates represent a highly vulnerable population requiring continuous care guidance following early postnatal discharge. This is due both to the fact that serious illnesses can present with vague symptoms at this age, and to parental inexperience and anxiety in distinguishing between normal neonatal characteristics and signs of illness.

Few studies have focused on neonatal admissions to PEDs; to our knowledge, this is the first study to describe the characteristics of an Italian neonatal population admitted to PEDs. Our comprehensive literature review identified eight relevant studies conducted in different countries (Table 4). Notably, all but two of these studies were retrospective, as is our study. Furthermore, all but one included fewer patients than our cohort, with the exception of a multicenter study conducted in the United States [15]. Across the studies, reasons for admission were similar, with neonatal jaundice being the most common reason, followed by benign gastrointestinal symptoms such as colic, upper airway inflammation, and skin rashes. The percentage of neonatal admissions among the overall pediatric population seen in PEDs was higher in our study compared to others (1.7% vs. 1.4%). However, the mean age of patients and the male‐to‐female ratio were consistent with other cohorts [16, 17].

The percentage of hospitalizations varied widely across the included studies. In our study, 25% of neonates required hospitalization, a rate similar to that observed in Turkish, English, and Portuguese cohorts. In contrast, neonates in the United States, Korea, and Singapore had higher hospitalization rates (47%–67%). These differences warrant further investigation, but one possible explanation is that in countries like Italy, Portugal, England, and Turkey, where PEDs admissions are free or fully covered by insurance, a higher proportion of neonates with non‐urgent conditions are seen in PEDs but do not require hospitalization [18, 19, 20, 21, 22, 23].

In our experience, the highest number of neonatal PED admissions occurred in August, while the highest number of hospitalizations was recorded in February. This trend may reflect the climatic conditions in Sicily, where hot summers can lead to dehydration, jaundice, and elevated body temperatures, while cold and windy winters may exacerbate respiratory conditions. Notably, the hospitalization rate among neonates was significantly higher than that of the general pediatric population admitted during the same period (25% vs. 11%), highlighting the increased vulnerability of neonatal patients.

All neonates assigned a “yellow” triage code required hospitalization, and 95% of those hospitalized had received this code at admission (Table 1). The hospitalization rate was significantly lower for neonates less than 7 days old (17%), consistent with findings from other studies. Both in the literature and in our study, the highest number of admissions occurred within the first 2 weeks of life. However, unlike other studies, we observed a progressive increase in hospitalizations during the third and fourth weeks of life.

Additionally, the majority of our patients were self‐referred (63%), with only 33% of these requiring hospitalization. In contrast, 67% of pediatrician‐referred patients required hospitalization, suggesting that many parental concerns may be unfounded. Our data highlight a significant gap in the continuity of care between hospitals and primary care services. The Sicilian “birth path” is designed to ensure continuity of care between birth centers and primary care pediatricians, with the goal of providing early care within the first week of life. However, in our study, 80% of neonates admitted to PEDs in the first week of life and 71.2% of those admitted in the second week were not under the care of a primary care pediatrician and were self‐referred to PEDs. This number decreased to 53% and 38% in the third and fourth weeks, respectively.

Our retrospective study cannot fully explain why such a significant proportion of neonates were not under primary care, but it is likely that the number of primary care pediatricians is insufficient to meet the needs of the entire pediatric population. Additionally, the bureaucratic processes involved in transitioning from hospital to community care may need to be simplified for families.

In summary, our findings, consistent with the limited available literature, indicate that the majority of neonatal PED admissions are due to non‐acute illnesses with benign symptoms that do not require immediate medical evaluation. This situation likely stems from insufficient caregiver knowledge and contributes to an unnecessary increase in workload for PED staff, which may distract from caring for truly urgent pediatric cases. It also exposes neonates and their families to hospital‐related risks such as infections and represents an unnecessary cost for the healthcare system. The underlying reasons for this issue may include a lack of awareness among families, inadequate education at the time of hospital discharge, and deficiencies in the primary care pediatric system. Our data underscore the need to enhance the role of primary care pediatricians, improve parental education on newborn care, and provide better community support during pregnancy and the immediate postnatal period to reduce unnecessary neonatal PED admissions and associated risks. Although neonatal PED admissions represent a small percentage of overall PED visits (1.7%), neonates are inherently vulnerable, and our data, which indicate a higher rate of neonatal admissions compared to other studies, suggest the need for neonatal‐oriented pathways in PEDs. This could include separate waiting areas and specialized personnel to reduce unnecessary waiting times and associated risks.

### 4.1 Limitations

The main limitation of this study is its retrospective design, which inherently limits the availability and completeness of certain data, particularly regarding perinatal, gestational, and familial history, which were often missing. Additionally, there is no standardized definition for distinguishing urgent from non‐urgent visits in the context of pediatric emergency care. The application of the Italian triage system, apart from the most obvious cases, remains somewhat subjective, potentially affecting the consistency of triage color assignments across different cases and hospitals.

## 5 Conclusions

Our findings, consistent with the limited literature available, confirm that the majority of neonatal admissions to PEDs are due to non‐acute illnesses. These admissions appear to be largely influenced by delays in transitioning care from hospitals to primary care pediatricians. Indeed, most of the neonates in our study were brought in by their parents, with only a minority being referred by pediatricians.

This study, which constitutes the first Italian registry of neonatal PED admissions and one of the largest studies on this topic globally, highlights a significant gap filled by parental inexperience and anxiety between the early postnatal discharge of newborns and the involvement of primary care providers. To address this issue, we recommend the development of educational training programs for parents, both before and after birth, to foster a stronger connection between families and primary care practitioners. These programs could help reduce unnecessary PED visits and improve the overall continuity of care for newborns.

## Ethics Statement

Ethics approval was granted by the Ethical Committee of the Medical Faculty of the University of Catania, Italy. Written informed consent was obtained from the parents of the patient.

## Consent

Written informed consent was obtained from the patient's parents for publication of this case report and the accompanying images.

## Conflicts of Interest

The authors declare no conflicts of interests.

## Data Availability

The data and materials of this case report are available from the corresponding author upon reasonable request.
